# Airway Management for an Adult Epiglottic Abscess

**DOI:** 10.7759/cureus.6771

**Published:** 2020-01-25

**Authors:** Panumart Manatpon, Ashleigh M Weyh, Conrad Gray, Saurin Shah, Jayanth Dasika

**Affiliations:** 1 Anesthesiology, University of Florida College of Medicine, Jacksonville, USA; 2 Oral and Maxillofacial Surgery, University of Florida Jacksonville, Jacksonville, USA; 3 Anesthesiology, University of Florida, College of Medicine, Jacksonville, USA

**Keywords:** epiglottitis, adult, airway management

## Abstract

Awake intubation is frequently described in the literature as the preferred method for securing the airway in adult patients with epiglottitis, whereas children with epiglottitis are usually intubated following an inhalational induction. However, if topicalization is difficult due to the presence of an abscess or an uncooperative patient, an inhalational induction may still be a reasonable approach in the adult patient. In a review of the literature, only one recent case report had been found describing an inhalational induction with video laryngoscopy. However, this attempt was unsuccessful, mandating the need for a surgical airway. Our case report describes a successful inhalational induction and video laryngoscope intubation without the use of a paralytic agent in an adult patient with an epiglottic abscess and moderate airway stenosis.

## Introduction

Acute epiglottitis is a life-threatening condition that requires prompt recognition and management. Largely due to the introduction of the Haemophilus influenzae type B vaccine in 1985, the annual incidence in children has decreased over the last several decades to less than one case per 100,000. In adults, however, the mean annual incidence per 100,000 adults has increased from 0.88 (from 1986-1990) to 3.1 (from 1996-2000) with a mortality of 7.1% [1]. Other causes of epiglottitis include polymicrobial infections as well as non-infectious causes such as trauma from foreign bodies, inhalation, and chemical burns [2]. Blood and throat cultures are often negative. 

Airway management in this patient population can be challenging to anesthesiologists because of the potential for complete airway obstruction leading to subsequent hypoxemia, cardiac arrest, and death. Children almost always have severe respiratory distress with fever, drooling, stridor, and “tripoding” (sitting and leaning forward in an attempt to optimize respiratory mechanics) and often will require immediate airway intervention. While tracheostomy used to be standard prior to 1968, inhalation induction with nasotracheal intubation is the more common method of securing the airway today. In addition, nasotracheal intubation may help prevent the risk of accidental extubation postoperatively, which could be disastrous [3]. 

Adults, on the other hand, can be more varied in presentation, and airway management is largely dependent on the severity of presentation. In mild-to-moderate cases without impending respiratory compromise, airway management varies from only medical treatment (antibiotics ± steroids) with vigilant monitoring to elective intubation in the operating room. Severe cases will require emergent airway intervention in 10.9% of the cases [4]. The presence of epiglottic abscess, stridor, and a history of diabetes mellitus are the most reliable clinical features associated with a need for airway intervention in adult patients with a diagnosis of epiglottitis [4]. A surgeon or anesthesiologist should always be prepared to perform a surgical airway if intubation is unsuccessful. 

Awake oral or nasal intubation is suggested as the preferred method of intubation in adults [3,5-7]. However, uncooperative patients may not tolerate an awake intubation. In addition, transtracheal and superior laryngeal nerve blocks may be dangerous in the presence of an abscess. Only two case reports have been found describing an asleep intubation with video laryngoscopy. One was unsuccessful, mandating the need for a surgical airway [8]. The second case was successful after an intravenous induction of propofol and succinylcholine. This was reported to be the first successful intubation of an adult patient with epiglottitis using video laryngoscopy [9]. However, we believe that the use of a paralytic agent and cessation of spontaneous ventilation is undesirable and potentially increases the risk of a surgical airway.

We report our airway management approach for a patient with acute epiglottitis presenting for direct laryngoscopy and drainage of an epiglottic abscess. Written consent was obtained from the patient for the publication of this case report. 

## Case presentation

A 33-year-old woman presented to the emergency department overnight with a one-day history of odynophagia, pharyngitis, dysphonia, dyspnea precipitated by lying down, hoarseness, drooling, and generalized malaise. Her medical history was significant for tobacco dependence, recent discovery of pregnancy, marijuana use (prior to pregnancy), and tonsillectomy as a child. She had not yet sought prenatal care. She had a carious, fractured tooth and recalled that a sharp piece had broken free and subsequently traumatized her throat as she swallowed it a few days prior. 

**Figure 1:**
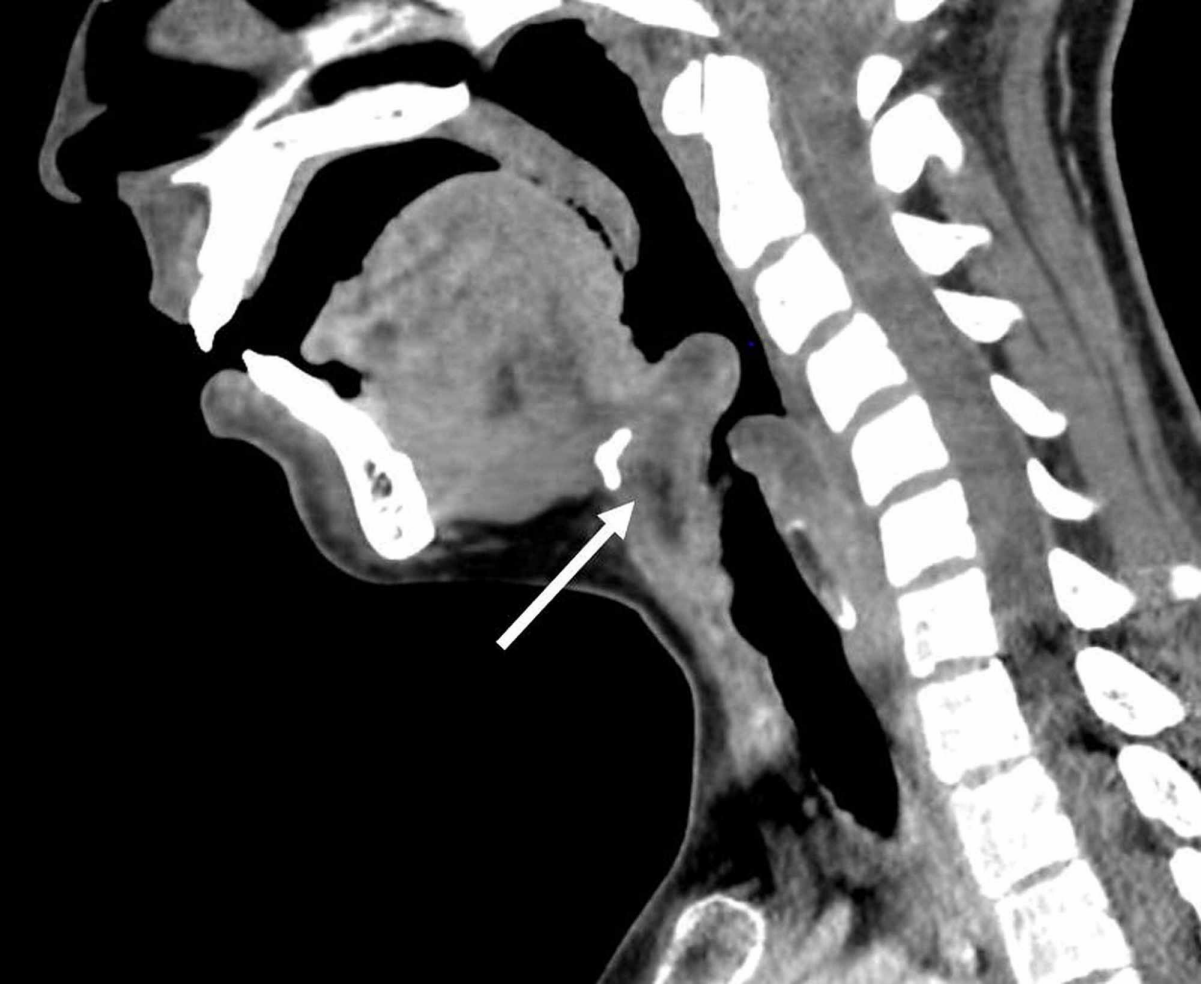
Sagittal computed tomography scan Sagittal computed tomography scan acquired in the emergency room, demonstrating edema of the epiglottis, 1.8 cm abscess formation, and airway compromise.

**Figure 2:**
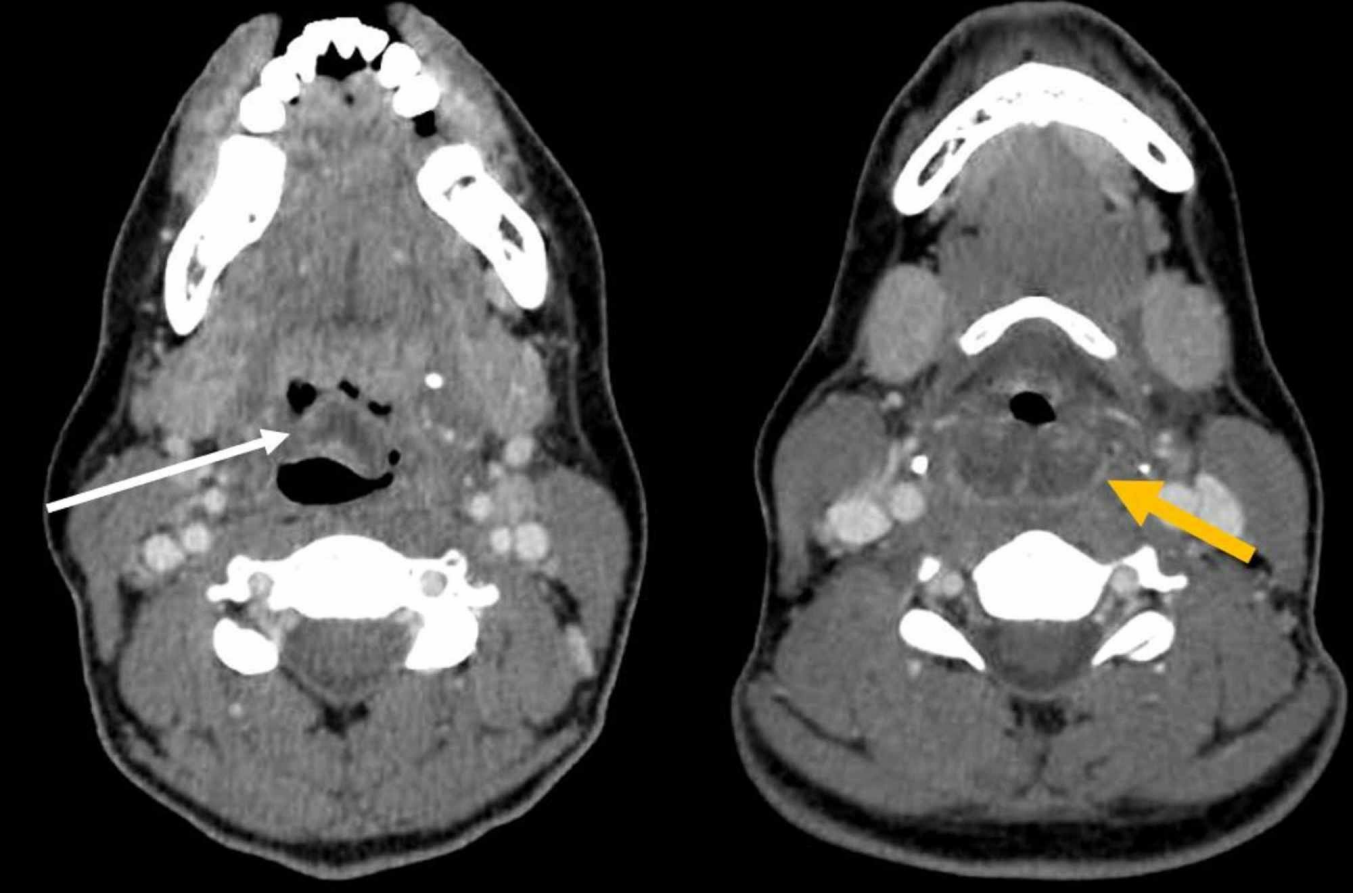
Axial computed tomography scans Axial computed tomography scans showing edema of the epiglottis (white arrow) and narrowing of the airway secondary to abscess and edema at the level of the hypopharynx (yellow arrow).

In the emergency department, she was afebrile (36.9°C), with a heart rate of 110 beats/min, a blood pressure of 115/61 mmHg, a respiratory rate of 22 breaths/min, and oxygen saturation of 99% on room air. On examination, she was sitting upright and was unable to swallow her saliva. Stridor was not appreciated upon auscultation of the lungs. The white blood cell count was elevated at 16,670/μL. To determine the etiology of her symptoms, a CT neck was performed and demonstrated markedly edematous change consistent with epiglottitis. It was also significant for a 1.8 cm fluid collection that involved the vallecula and epiglottis, with diffuse edema of the epiglottis and aryepiglottic folds extending posterior into the hypopharynx. This caused moderate airway stenosis, most prominent at the aryepiglottic folds (Figures 1, 2). 

The provisional diagnosis of acute epiglottitis with a developing epiglottic abscess was made, and the oral and maxillofacial surgery and obstetric departments were consulted. Ultrasonography performed by the obstetric team showed a 12-week single intrauterine pregnancy with a fetal heart rate of 162 beats/min. As this was still the first trimester, no further pregnancy testing or management was planned except for confirming postoperative fetal heart tones. Clindamycin and dexamethasone were administered intravenously, and the patient was made nil per os. She was scheduled for a direct laryngoscopy and exploration in the operating room the next morning. 

On examination in the preoperative area, the patient’s condition had improved overnight following antibiotic and steroid administration, as she was now able to tolerate lying flat. Airway examination revealed a Mallampati score of II, thyromental distance > 6 cm, two missing teeth, an adequate mouth opening, and no limitations to neck movement. She had significant pain on anterior neck palpation with prominent cervical lymphadenopathy. She was not visibly drooling; however, she required frequent suctioning. 

**Figure 3:**
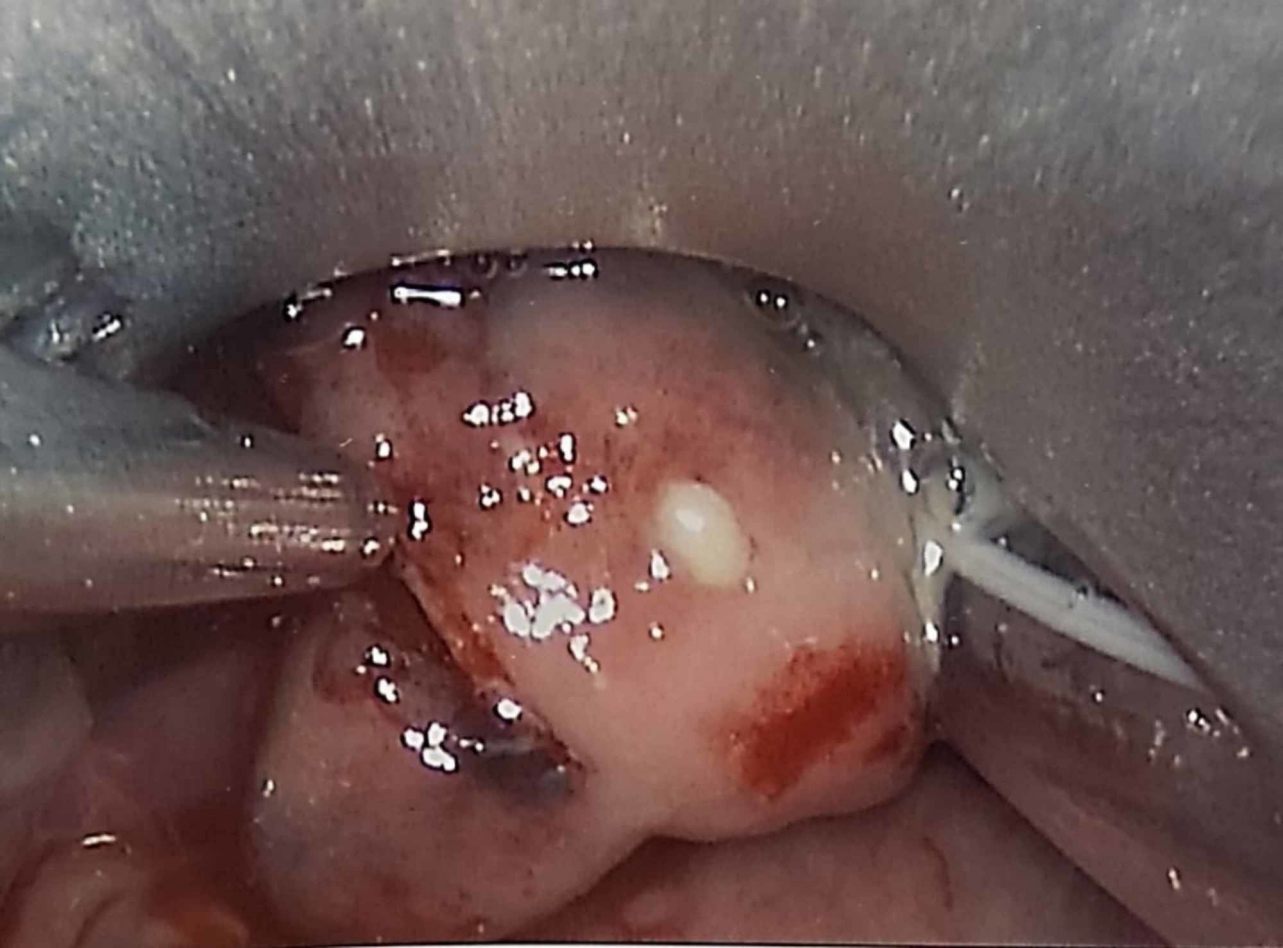
Intraoperative image Intraoperative images during direct laryngoscopy, demonstrating severe edema of the epiglottis and spontaneously draining abscess.

The plan for securing the airway was deep inhalational induction maintaining spontaneous ventilation followed by video laryngoscopy. As a backup, a fiberoptic scope was available and the surgical team was prepared with an emergent tracheostomy kit. In the operating room, the patient was positioned sitting up with standard American Society of Anesthesiologist monitors. A 4% lidocaine nebulizer was given to blunt airway reactivity, and the patient was preoxygenated with 100% O_2_. Next, an inhalation induction with 8% sevoflurane was initiated. The patient was then repositioned to the supine position. After confirming loss of lid reflex while maintaining spontaneous ventilation, videolaryngoscopy was performed which demonstrated marked swelling of the epiglottis with purulent discharge (Figure 3). A 7.0 endotracheal tube was successfully passed into the trachea. No paralytic agent was used during the intubation in order to maintain spontaneous ventilation. 

The epiglottic abscess was biopsied and drained by the surgical team followed by an uneventful extubation after demonstration of a cuff leak. Postoperatively, the patient reported significant improvement of symptoms. Repeat ultrasonography showed fetal movement with a heart rate of 153 beats/min. She was discharged home on postoperative day 2. Blood cultures from initial presentation remained negative for five days. No specific organisms were found from cultured materials intraoperatively. 

## Discussion

Airway intervention in epiglottitis is more common in children than adults [3]. In fact, adults without severe features of epiglottitis can often be medically managed with careful monitoring, antibiotics, and steroids. Although our patient did not have stridor or signs of impending respiratory failure, airway intervention was required to drain the epiglottic abscess. While an awake intubation is often recommended for adult patients with epiglottitis [3,5,6], it was thought that topicalization would be challenging in our patient with severe pharyngitis, placing her at risk for laryngospasm and complete airway obstruction. Direct airway blocks (i.e., superior and/or recurrent laryngeal) could potentially have led to abscess rupture and difficulty visualizing the airway with any non-surgical technique. A deep inhalational induction without the use of a paralytic agent provided the benefit of maintaining spontaneous ventilation while also keeping the patient comfortable under general anesthesia prior to securing the airway. We acknowledge that an oral or nasal approach with the fiberoptic endoscope may still have been reasonable in this patient. It may also have been reasonable to induce the patient with intravenous ketamine in order to maintain spontaneous ventilation.

## Conclusions

Adult epiglottic abscess can be life-threatening, and presents unique difficulties when intubation is necessary. In a patient not yet experiencing severe respiratory compromise, inhalational induction and video laryngoscopy may be a reasonable approach to securing the airway. 
